# A Genome-Wide Association Study Finds Genetic Associations with Broadly-Defined Headache in UK Biobank (N = 223,773)

**DOI:** 10.1016/j.ebiom.2018.01.023

**Published:** 2018-01-31

**Authors:** Weihua Meng, Mark J. Adams, Harry L. Hebert, Ian J. Deary, Andrew M. McIntosh, Blair H. Smith

**Affiliations:** a Division of Population Health Sciences, School of Medicine, University of Dundee, Dundee DD2 4BF, UK; b Division of Psychiatry, Edinburgh Medical School, University of Edinburgh, Edinburgh EH10 5HF, UK; c Centre for Cognitive Ageing and Cognitive Epidemiology, Department of Psychology, University of Edinburgh, Edinburgh EH8 9JZ, UK

**Keywords:** Headache, Genome-wide association study, *LRP1*, UK biobank, Tissue expression

## Abstract

**Background:**

Headache is the most common neurological symptom and a leading cause of years lived with disability. We sought to identify the genetic variants associated with a broadly-defined headache phenotype in 223,773 subjects from the UK Biobank cohort.

**Methods:**

We defined headache based on a specific question answered by the UK Biobank participants. We performed a genome-wide association study of headache as a single entity, using 74,461 cases and 149,312 controls.

**Results:**

We identified 3343 SNPs which reached the genome-wide significance level of *P* < 5 × 10^− 8^. The SNPs were located in 28 loci, with the top SNP of rs11172113 in the *LRP1* gene having a *P* value of 4.92 × 10^− 47^. Of the 28 loci, 14 have previously been associated with migraine. Among 14 new loci, rs77804065 with a *P* value of 5.87 × 10^− 15^ in the *LINC02210-CRHR1* gene was the top SNP. Significant relationships between multiple brain tissues and genetic associations were identified through tissue expression analysis. We also identified significant positive genetic correlations between headache and many psychological traits.

**Conclusions:**

Our results suggest that brain function is closely related to broadly-defined headache. In addition, we found that many psychological traits have genetic correlations with headache.

## Highlights

• This genome-wide association study identified 28 genomic loci for broadly-defined headache, among which, 14 are new.
• Through tissue expression analysis, brain tissues showed significant relationships to broadly-defined headache.
• Broadly-defined headache shared common genetic components with many psychological traits such as neuroticism.

This genetic study using the UK Biobank resource has identified 28 genomic loci for broadly-defined headache, among which, 14 are new loci. In addition, it has provided extra evidence that the functions of brain tissues are closely related to headache. Moreover, it has suggested that headache and many psychological disorders share common genetic factors. These findings will not only contribute to the understanding of the causes of headache (and its subtypes) and its relationships with psychological disorders, it might also bring potential genetic targets for drug treatment for patients with headache and psychological disorders.

## 1 Introduction

Headache is the most common neurological symptom, with a life time prevalence of over 90% in the general population in the UK (Boardman et al., 2003). It represents 4.4% of consultations in primary care and 30% of outpatient consultations in neurology (Larner, 2006, Latinovic et al., 2006).

According to the International Headache Society, headache can be generally divided into two categories: primary headache, if not associated with another disorder; and secondary headache if associated with an underlying medical illness (Headache Classification Committee of the International Headache Society, 2013). Primary headaches mainly include migraine, tension-type, and cluster headaches. Secondary headaches include any head pain caused by infection, neoplasm, head injury, some metabolic disorders, or drugs (Headache Classification Committee of the International Headache Society, 2013).

In a comprehensive review of population-based epidemiological studies of headache, the global prevalence of recurrent headache in all ages was found to be 46% for all headaches, including 11% for migraine and 42% for tension-type headache (Stovner et al., 2007). Tension-type headache is the most prevalent type of headache, whereas migraine is the most disabling (Riesco et al., 2007).

Migraine affects around 6 million people in England in the age range 16–65 and it is the sixth cause in terms of years of life lost to disability according to the Global Burden of Diseases 2013 (Global Burden of Disease Study 2013 Collaborators, 2015). Migraine costs the National Health Service almost £2 billion per year (Steiner et al., 2003). It presents with recurrent headache attacks and/or hypersensitivity to light and sound. Around one third of migraineurs experience an aura, which are transient neurological symptoms mostly involving the visual system (Silberstein, 2004).

Family studies and twin studies have suggested that both migraine and tension-type headache are heritable traits with a heritability over 40% (Russell et al., 2007, Schürks, 2012). Recently, genome-wide association studies (GWAS) have identified many genetic loci associated with migraine (Anttila et al., 2010, Anttila et al., 2013, Chasman et al., 2011, Freilinger et al., 2012, Ligthart et al., 2011). A GWAS meta-analysis of 375,000 patients involving 22 centers has identified 38 genetic susceptibility loci for migraine with the *LRP1* region in chromosome 12 being the most strongly associated (Gormley et al., 2016a). Along with other GWAS on migraine, the total number of loci identified to be associated with migraine is currently 47 (Gormley et al., 2016b). No GWAS have been performed for tension-type headache so far.

There are several phenotypic associations between headache and metabolic, psychological, and other factors such as obesity (Scher et al., 2003, Waldie and Poulton, 2002). Genome-wide association studies provide a potential route to discover genetic correlations with other complex traits and diseases that in turn may provide clues to shared genetic architectures and etiologies (Bulik-Sullivan et al., 2015a).

To identify the genetic variants associated with headache, we conducted this GWAS using the UK Biobank cohort which has never been contributed to genetic studies of headache including migraine. We used a broadly-defined headache phenotype, the one available in the UK Biobank dataset. Secondly, we sought to test for shared genetic associations with other complex traits and diseases using linkage-disequilibrium score regression (Bulik-Sullivan et al., 2015b).

## 2 Materials and Methods

### 2.1 Participants and Genetic Information of Participants

The UK Biobank is a health research resource that aims to improve the prevention, diagnosis and treatment of a wide range of illnesses. The UK Biobank cohort recruited over 500,000 people aged between 40 and 69 years in 2006–2010 across the UK. Participants completed a detailed clinical, demographic, and lifestyle questionnaire, underwent clinical measures, provided biological samples (blood, urine and saliva) for future analysis, and agreed to have their health records accessed. The informed consent of all participants has been obtained. Details of the UK Biobank resource can be found at www.ukbiobank.ac.uk. UK Biobank received ethical approval from the National Health Service National Research Ethics Service (reference 11/NW/0382). The current analyses were conducted under approved UK Biobank data application number 4844.

The detailed methods of DNA extraction and quality control can be found at http://www.ukbiobank.ac.uk/wp-content/uploads/2014/04/DNA-Extraction-at-UK-Biobank-October-2014.pdf. Participants' DNA was genotyped by bespoke Affymetrix UK Biobank chips. Standard QC steps were performed by the Wellcome Trust Centre for Human Genetics at Oxford University. The detailed QC steps can be found at http://biobank.ctsu.ox.ac.uk/crystal/refer.cgi?id=155580.

In July 2017, the genetic information (including directly genotyped genotypes and imputed genotypes) from 501,708 samples was released to UK Biobank project research collaborators. The detailed QC steps of imputation are described by Bycroft et al. (Bycroft et al., 2017).

### 2.2 Phenotypic Information on Pain

The UK Biobank participants were offered a pain-related questionnaire, which included the question: ‘in the last month have you experienced any of the following that interfered with your usual activities?’. The options were: 1. Headache; 2. Facial pain; 3. Neck or shoulder pain; 4. Back pain; 5. Stomach or abdominal pain; 6. Hip pain; 7. Knee pain; 8. Pain all over the body; 9. None of the above; 10. Prefer not to say. Participants could select more than one option. (UK Biobank Questionnaire field ID: 6159).

The headache cases in this study were those who selected the ‘Headache’ option for the above question, regardless of whether they had selected other options.

The controls in this study were those who selected the ‘None of the above’ option.

### 2.3 Statistical Analysis

In this study, we used BGENIE (https://jmarchini.org/bgenie/) to be the main GWAS software and removed single nucleotide polymorphism (SNPs) with INFO scores < 0.1, with minor allele frequency < 0.5%, or those that failed Hardy-Weinberg tests *P* < 10^− 6^. SNPs on the X and Y chromosomes and mitochondrial SNPs as well as imputed SNPs that were not in the Haplotype Reference Consortium panel were excluded from analyses. Standard Frequentist association tests using BGENIE was used to perform association studies adjusting for age, sex, body mass index (BMI), 9 population principal components, genotyping arrays, and assessment centers. Gender difference between cases and controls was compared using chi-square testing. Age and BMI were compared using independent t testing in IBM SPSS 22 (IBM Corporation, New York). SNP associations were considered significant if they had a *P* value < 5 × 10^− 8^. GCTA was used to calculate SNP-based or narrow-sense heritability (Yang et al., 2011) using a genomic relationship matrix calculated from genotyped autosomal SNPs.

SNP functional annotations were applied by the FUMA web application and a Manhattan plot was generated by R (Watanabe et al., 2017). R was also used to generate the corresponding Q-Q plot, a tool to evaluate differences between cases and controls caused by potential confounders.

The gene analysis and gene-set analysis were performed with MAGMA v1.6, which was integrated in FUMA (de Leeuw et al., 2015). Both analyses were based on GWAS summary statistics. In gene analysis, summary statistics of SNPs are aggregated to the level of whole genes, testing the joint association of all SNPs in the gene with the phenotype. In gene-set analysis, individual genes are aggregated to groups of genes sharing certain biological, functional or other characteristics. This will provide insight into the involvement of specific biological pathways or cellular functions in the genetic etiology of a phenotype. Tissue expression analysis was obtained from GTEx (https://www.gtexportal.org/home/) which was also integrated in FUMA. The purpose of using FUMA web application was to provide extra information to visualize and interpret GWAS results.

In order to identify genetic correlations between headache and other complex traits, we used linkage disequilibrium score regression through LD Hub v1.4.1 (available at http://ldsc.broadinstitute.org/ldhub/) (Zheng et al., 2017). This web-tool uses individual SNP allele effect sizes and the average linkage disequilibrium in a region to estimate the bivariate genetic correlations of headache with 234 traits. Those with *P* values of 2.1 × 10^− 4^ (0.05/234) or less should be regarded as surviving Bonferroni adjustment for multiple testing.

## 3 Results

### 3.1 GWAS Results

During the initial assessment visit (2006–2010), at which participants were recruited and consent was given by 501,708 UK Biobank participants, the specific pain question (see the Materials and Methods section for details) received 775,252 responses to all options. Among these responses, the number of participants who selected the ‘Headache’ option was 102,994 (cases), and the number of participants who selected the ‘None of the above’ option was 197,149 (controls). We further removed those whose ancestry was not white British (*n* = 22,694) based on principal component analysis, those who were related to one or more others in the cohort (a cut-off value of 0.025 in the generation of the genetic relationship matrix) (n = 52,166), those who were also participants in a Psychiatric Genomics Consortium Major Depressive Disorder cohort (n = 597), and those who failed quality-control (QC) (n = 913). Thus we finally identified 74,461 cases (27,350 males and 47,111 females) and 149,312 controls (71,480 males and 77,832 females) for the GWAS association analysis. After quality control, there were 9,304,965 SNPs for the GWAS analysis.

The clinical characteristics of these cases and controls are summarized in Table 1. There were statistical differences (*P* < 0.001) in age, sex and BMI between cases and controls.

**Table 1:** Clinical characteristics of headache cases (74,461) and controls (149,312).

Covariates	Cases	Controls	*P*^b^
Sex (male:female)	27,350: 47,111	71,480: 77,832	< 0.001
Age (years)	54.38 (7.95)	56.93 (7.97)	< 0.001
BMI^a^ (kg/m^2^)	27.50 (5.05)	26.66 (4.30)	< 0.001

Continuous covariates were presented as mean (standard deviation).

a BMI: body mass index.

b A chi-square test was used to test the difference of gender frequency between cases and controls and an independent *t*-test was used for other covariates.

**Fig. 1:**
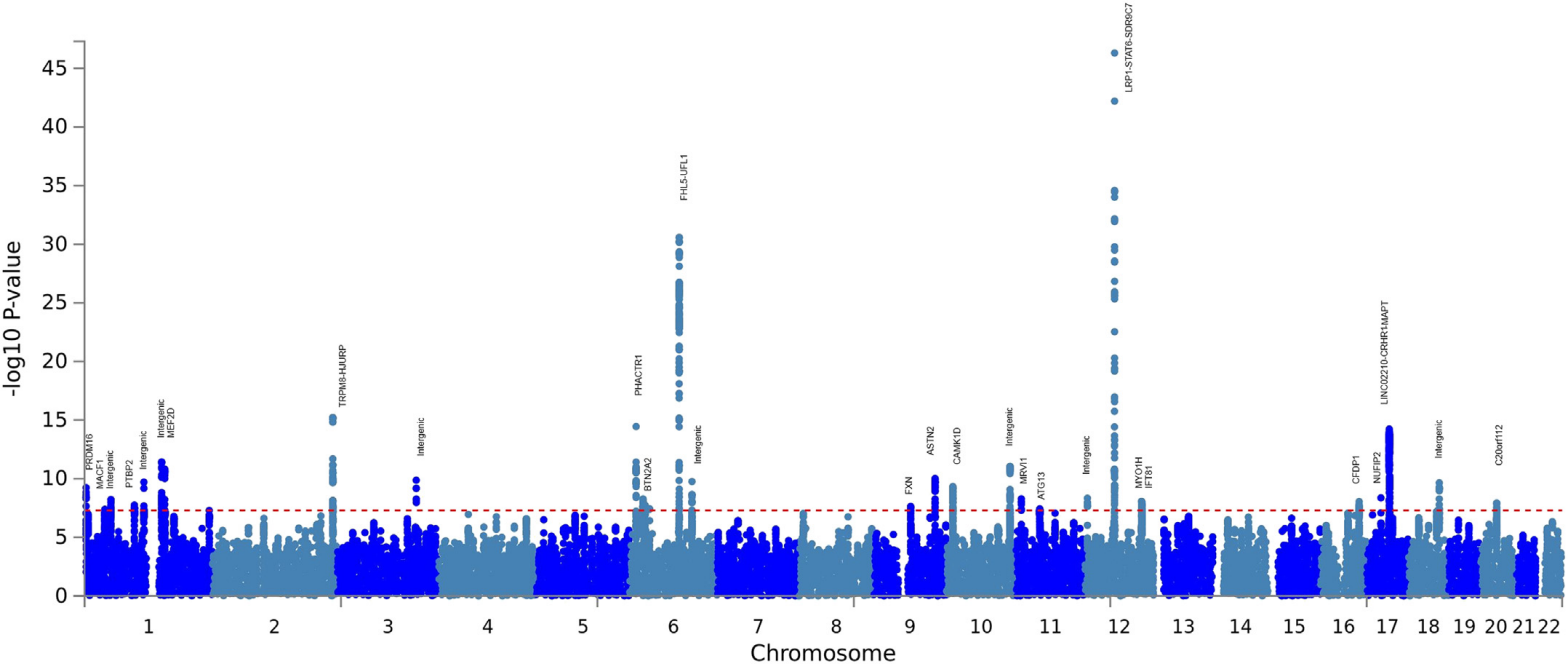
The Manhattan plot of the GWAS on headache using the UK Biobank cohort.

We identified 3343 SNPs which reached GWAS significance of *P* < 5 × 10^− 8^ (Fig. 1, Supplementary Table 1). These SNPs represented 28 independent loci including 14 newly-identified loci (Table 2).

**Table 2:** Summary of the 28 loci associated with broadly-defined headache.

Locus rank	Gene	Chromosome	Lead SNP	*P*	Effective allele	MAF^a^	Beta	SE^b^	New loci?
1	*LRP1-STAT6-SDR9C7*	12	rs11172113	4.92 × 10^− 47^	C	0.38	− 0.0202	0.0014	
2	*FHL5-UFL1*	6	rs9486715	2.58 × 10^− 31^	C	0.35	0.0171	0.0015	
3	*TRPM8-HJURP*	2	rs2362290	6.25 × 10–^16^	A	0.17	− 0.0141	0.0017	
4	*PHACTR1*	6	rs9349379	3.59 × 10^− 15^	G	0.40	− 0.0110	0.0014	
5	*LINC02210-CRHR1-MAPT*	17	rs77804065	5.87 × 10^− 15^	T	0.25	0.0130	0.0017	New locus
6	*Intergenic*	1	rs12740679	3.66 × 10^− 12^	G	0.26	0.0110	0.0016	New locus
7	*Intergenic*	10	rs78438709	8.57 × 10^− 12^	G	0.07	− 0.0186	0.0027	
8	*MEF2D*	1	rs1050316	1.54 × 10^− 11^	T	0.33	− 0.0098	0.0015	
9	*ASTN2*	9	rs17220352	9.42 × 10^− 11^	G	0.25	0.0104	0.0016	
10	*Intergenic*	3	rs34097149	1.33 × 10^− 10^	C	0.03	− 0.0297	0.0046	
11	*Intergenic*	6	rs9490318	1.72 × 10^− 10^	T	0.14	0.0124	0.0019	
12	*Intergenic*	1	rs12134493	1.93 × 10^− 10^	A	0.11	0.0136	0.0021	
13	*Intergenic*	18	rs4941139	2.22 × 10^− 10^	C	0.32	0.0093	0.0015	New locus
14	*CAMK1D*	10	rs2895526	4.61 × 10^− 10^	A	0.48	− 0.0086	0.0014	New locus
15	*PRDM16*	1	rs56304645	5.74 × 10^− 10^	T	0.26	0.0102	0.0016	
16	*NUFIP2*	17	rs8614	4.25 × 10^− 9^	A	0.19	0.0105	0.0018	New locus
17	*Intergenic*	12	rs10774231	4.55 × 10^− 9^	C	0.42	− 0.0082	0.0014	
18	*MRVI1*	11	rs4909945	5.13 × 10^− 9^	C	0.30	0.0087	0.0015	
19	*BTN2A2*	6	rs2072806	5.30 × 10^− 9^	G	0.12	− 0.0121	0.0021	New locus
20	*Intergenic*	1	rs7555006	5.87 × 10^− 9^	G	0.44	0.0081	0.0014	New locus
21	*MYO1H*	12	rs6606710	8.44 × 10^− 9^	C	0.42	0.0084	0.0015	New locus
22	*IFT81*	12	rs7300001	8.86 × 10^− 9^	G	0.10	− 0.0144	0.0025	New locus
23	*NOL4L*	20	rs1555132	1.16 × 10^− 8^	A	0.34	0.0083	0.0015	New locus
24	*CFDP1*	16	rs1011121	1.46 × 10^− 8^	G	0.43	− 0.0080	0.0014	
25	*PTBP2*	1	rs3748784	1.75 × 10^− 8^	G	0.47	− 0.0078	0.0014	New locus
26	*FXN*	9	rs4596713	2.30 × 10^− 8^	T	0.41	− 0.0078	0.0014	New locus
27	*ATG13*	11	rs56349329	3.69 × 10^− 8^	A	0.16	− 0.0104	0.0019	New locus
28	*MACF1*	1	rs2036465	4.01 × 10^− 8^	C	0.20	− 0.0093	0.0017	New locus

a MAF: minor allele frequency.

b SE: standard error.

**Supplementary Fig. 1:**
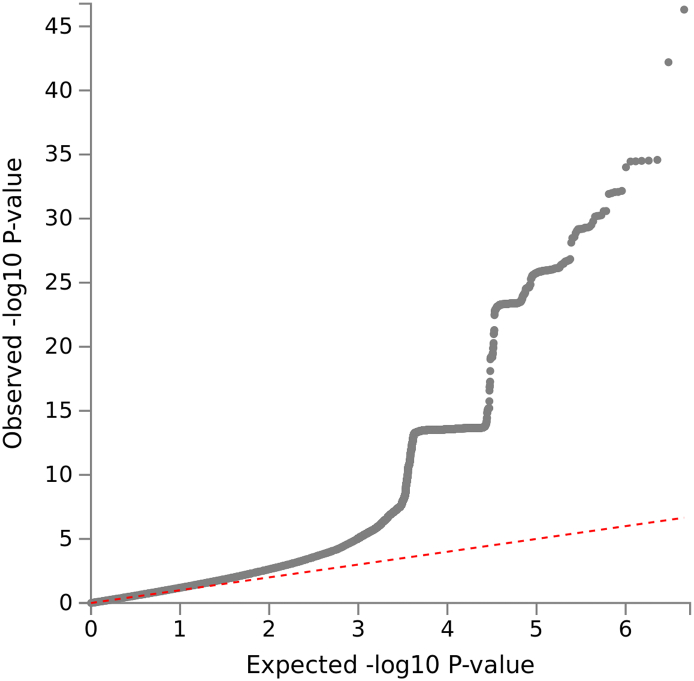
The Q-Q plot of the GWAS on headache using UK Biobank.

The first cluster was in the LDL receptor related protein 1 (*LRP1*) gene in the chromosome 12 area with a lowest *P* value of 4.92 × 10^− 47^ for rs11172113 (C allele, odds ratio (OR): 0.98). The second cluster was in the four and a half LIM domains 5 (*FHL5*) gene in chromosome 6 with a lowest *P* value of 2.58 × 10^− 31^ for rs9486715 (C allele, OR: 1.02). Among the 28 loci, 14 of them were newly identified. Rs77804065 in the LINC02210-CRHR1 readthrough (*LINC02210-CRHR1*) gene with a *P* value of 5.87 × 10^− 15^ (T allele, OR: 1.01) was the most strongly associated among the newly-identified loci. The Q-Q plot of the GWAS is shown in the Supplementary Fig. 1. The SNP-based heritability of broadly-defined headache was 0.211 (standard error = 0.015).

### 3.2 Gene Analysis, Gene-Set Analysis and Tissue Expression Analysis by FUMA

In the gene analysis by MAGMA integrated in FUMA, all the SNPs are mapped to 19,436 protein coding genes if the SNPs are located within genes. The default SNP-wise (mean) model for the gene analysis was applied. The signal transducer and activator of transcription 6 (*STAT6*) gene reached the lowest *P* value of 1.1 × 10^− 46^, followed by UFM1 specific ligase 1 (*UFL1*) (*P* = 2.53 × 10^− 26^), *FHL5* (*P* = 8.64 × 10^− 25^) and *LRP1* (*P* = 3.85 × 10^− 23^). All genes (*N* = 160) with a *P* < 3 × 10^− 6^ (0.05/19436) are included in the Supplementary Table 2.

In the gene set analysis by MAGMA integrated in FUMA, a total of 10,894 gene sets were tested and a default competitive test model was applied. Gene sets of positive regulation of gene expression, positive regulation of transcription from RNA polymerase ii promoter, neurogenesis, and excitatory synapse reached a *P* value < 0.0001, but not statistical significant of *P* < 5 × 10^− 6^ (0.05/10,894). The top 10 gene sets from the analysis were included in the Supplementary Table 3.

**Fig. 2:**
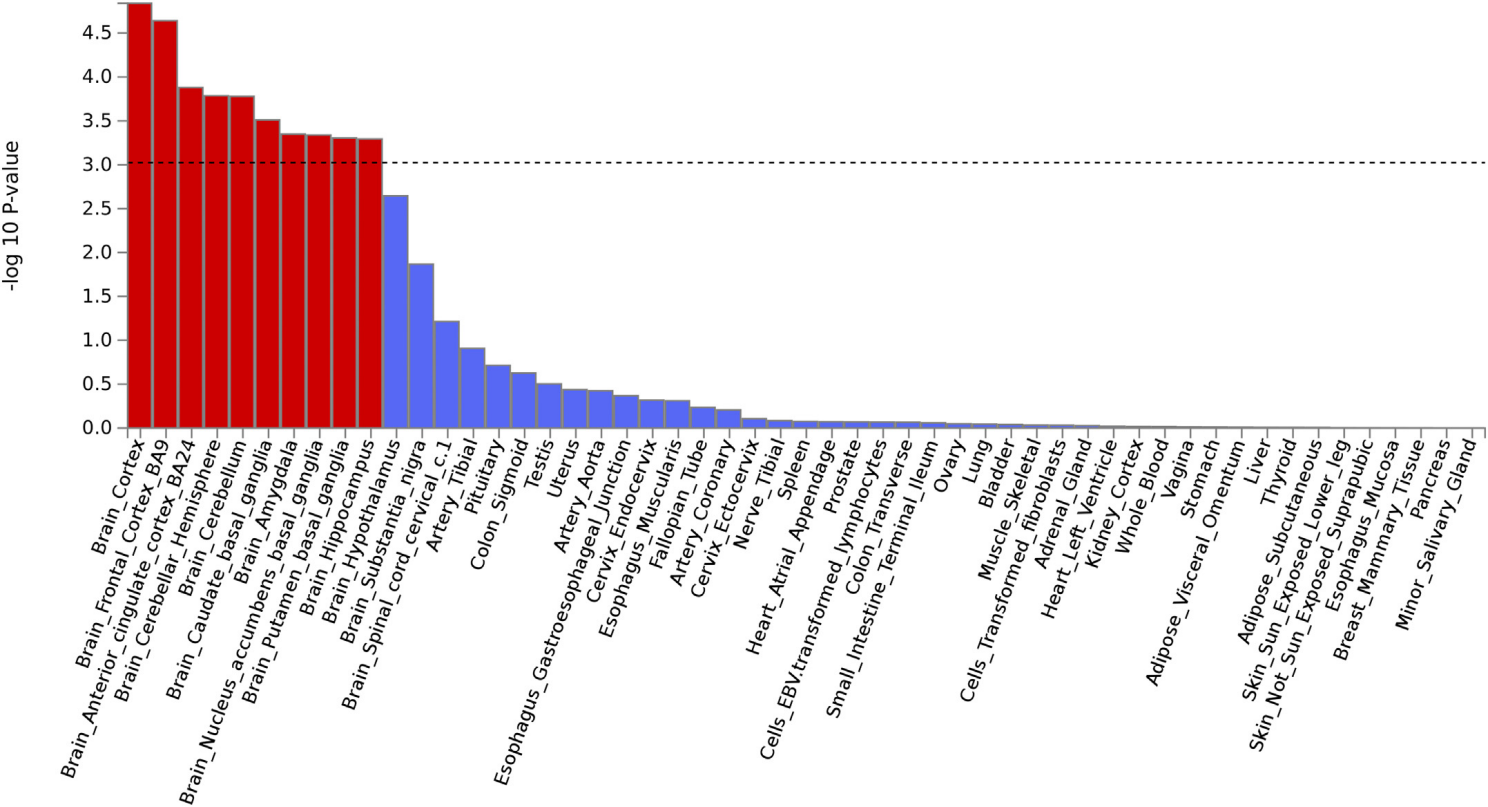
The tissue expression results on 53 specific tissue types by GTEx in FUMA.

In the tissue expression analysis by GTEx integrated in the FUMA, average gene-expression per tissue type was used as gene covariate to test positive relationship between gene expression in a specific tissue type and genetic associations. Two types of tissue analysis were performed. One used 30 general tissue types and the other used 53 specific tissue types. In the expression analysis of 30 general tissue types, the tissue in the brain showed the most significant *P* value (*P* = 4.12 × 10^− 6^), followed by the tissue from blood vessel (*P* = 0.014) (Supplementary Table 4). In the 53 specific tissue types, tissues from the brain cortex reached a lowest *P* value of 1.44 × 10^− 5^ and most of the brain specific tissues also reached a significant *P* value of 0.001 (0.05/53). See Fig. 2 and Supplementary Table 5.

### 3.3 Genetic Correlation Analysis by LD Hub

Through the genetic correlation analysis, we identified multiple significant positive correlations for headache (Supplementary Table 6). The significant genetic correlations (rg) surviving multiple testing correction (*P* < 0.05/234) were: neuroticism (rg = 0.50, *P* = 2.24 × 10^− 72^), depressive symptoms (rg = 0.52, *P* = 1.60 × 10^− 46^), years of education (rg = − 0.28, *P* = 5.25 × 10^− 37^), maternal age at first delivery (rg = − 0.32, *P* = 3.97 × 10^− 29^), subjective wellbeing (rg = − 0.37, *P* = 9.51 × 10^− 19^), insomnia (rg = 0.42, *P* = 2.54 × 10^− 18^), and major depressive disorder (rg = 0.39, *P* = 1.57 × 10^− 11^).

## 4 Discussion

We have performed a GWAS on broadly-defined headache as a single entity using the UK Biobank resource and found that variants in 28 loci were associated with having experienced headache within the last month to the extent that it interfered with usual activities. Evidence from tissue expression analysis showed that brain function is closely related to this broadly-defined headache. In addition, we found that headache was genetically correlated with a number of psychological factors, including those linked to a higher tendency toward experiencing negative emotional states, and shorter duration of education.

In this study, we defined headache cases and controls based on the responses by UK Biobank participants to a specific pain question. This question focused on headache occurrence, sufficient to cause interference with activities, during the previous month. We can therefore only treat headache as a global condition to perform GWAS. We can hypothesize, based on the reported population prevalence of each subtype (Stovner et al., 2007, Riesco et al., 2007), that tension-type headache will be the most common diagnosis among cases, followed by migraine, and that many will have experienced more than one type of headache. Whether the SNPs identified are associated with one or more of these diagnoses specifically, or with headache globally remains unclear until further research is conducted. It is important to note that half of the 28 loci were previously reported loci for migraine (Table 2).

The UK Biobank genetic resource is especially useful as a screening tool to test whether heterogeneous phenotypes such as headache have genetic components at all, as the UK Biobank has collected many heterogeneous phenotypes which need further genetic investigation. An example of such a GWAS approach was adopted by Deary et al. on self-reported tiredness (a heterogeneous phenotype) using the UK Biobank cohort (Deary et al., 2016). GWAS like this will help with genetic stratification analysis for heterogeneous phenotypes. This genetic discovery phase could be part of the partitioning or classification of broadly-reported or broadly-defined headache, improving our understanding of its etiology, diagnosis, prognosis and the development of treatments.

The findings of this research will facilitate the next essential phases of research into the genetics and causes of headache. They will enable investigators to conduct adequate power calculations for future studies and will provide candidate loci to examine in replication studies. Even with our very heterogeneous group of cases, including many different headache subtypes, we were still able to identify numerous GWAS associations. Therefore, it can be expected that in a GWAS of a more intensively-phenotyped headache cohort or a subtype of headache such as tension-type headache for which no GWAS has yet been performed, genetic contributions could be more distinctive and easier to identify than in this study (for example, GWAS hits can be identified using fewer samples). Since our results are of GWAS significance, the genes (such as *LRP1* and *FHL5*) identified for migraine by other GWAS are also promising candidate genes for tension-type headache. The majority of the patients with migraine (over 90%) also suffer from tension-type headache (Lyngberg et al., 2005), and it is possible that migraine and tension-type headache might share common genetic components.

In this GWAS, we have identified 28 loci for headache. One was in the *LRP1* gene with a lowest *P* value of 4.92 × 10^− 47^ for rs11172113. The *LRP1* gene encodes a precursor protein that is processed by furin in the trans-Golgi complex, generating a 515 kDa alpha-chain and an 85 kDa beta-chain associated non-covalently (Lillis et al., 2008). This protein is involved in multiple cellular processes, including intracellular signaling, lipid homeostasis, and clearance of apoptotic cells (Etique et al., 2013). The *LRP1* gene has also been reported to be associated with Alzheimer's disease, cardiovascular disease, and tumors (Kanekiyo and Bu, 2014, Llorente-Cortés and Badimon, 2005, Song et al., 2009). Chasman et al. first identified the association between the *LRP1* gene and migraine in a Caucasian population (Chasman et al., 2011). The association was then replicated by Freilinger et al. in migraine without aura in German and Dutch patients (Freilinger et al., 2012). The locus was further replicated in Indian but not in Chinese populations (Fan et al., 2014, Ghosh et al., 2013). It is worth noting that many nearby supporting SNPs for the *LRP1* region were located in the nearby gene of *STAT6* (which was also strongly associated with headache).

The second SNP cluster was in the *FHL5* gene area with a lowest *P* value of 2.58 × 10^− 31^ for rs9486715. The protein encoded by this gene is expressed with activator of cAMP-responsive element modulator (CREM) (Fimia et al., 1999). It is associated with CREM and confers a powerful transcriptional activation function (Fimia et al., 1999). The locus was first reported by Anttila et al. to be associated with migraine (Anttila et al., 2013). Although replication failed in a Chinese population, the locus was replicated by Gormley et al. in a large meta-analysis on migraine (Gormley et al., 2016a, Lin et al., 2015). Both the *LRP1* and *FHL5* genes are also candidate genes for cervical artery dissection suggesting vascular involvement in headache (Gormley et al., 2016b). Just as the *LRP1* region extends to the *STAT6* gene, the *FHL5* cluster extends to the *UFL1* gene.

The strongest association among the 14 newly proposed loci is located in the *LINC02210-CRHR1* gene. The corticotropin releasing hormone receptor 1 (*CRHR1*) gene has been reported to be associated with stress, panic disorder, neuroticism (Henckens et al., 2016, Weber et al., 2016, DeYoung et al., 2011). This is matched with our genetic correlation results by LD hub suggesting headache and psychological traits share genetic architectures (see Discussion below). The locus extends to the microtubule associated protein tau (*MAPT*) gene region. Other new identified loci included: calcium/calmodulin dependent protein kinase ID (*CAMK1D), NUFIP2, FMR1 interacting protein 2 (NUFIP2), butyrophilin subfamily 2 member A2 (BTN2A2), myosin IH (MYO1H), intraflagellar transport 81 (IFT81), nucleolar protein 4 like (NOL4L), polypyrimidine tract binding protein 2 (PTBP2), frataxin (FXN), autophagy related 13 (ATG13), microtubule-actin crosslinking factor 1 (MACF1)* and some intergenic regions. It is interesting to notice that GWAS have identified that *CAMK1D* and *MACF1* are involved in vascular disorders (hypertension and peripheral artery disease) (Frau et al., 2014, Ward-Caviness et al., 2016), again supporting a vascular contribution to headache.

It is also interesting to note that the *STAT6* gene was the most significant in the gene analysis with headache and not the *LRP1* gene where the top SNP resides. These two genes are next to each other in the genome and have previously been associated with disorders related to the immune system such as food allergen sensitization and Sjogren's Disease (Hancock et al., 2012, Johar et al., 2015). It is reported that IL4/STAT6 signaling activates neural stem cell proliferation and neurogenesis in zebrafish brain, which indicates the importance of the gene in neuron function (Bhattarai et al., 2016). The gene sets analysis revealed that genes involved in the neurogenesis are associated with headache which is consistent with the tissue expression analysis.

Both tissue expression analysis on 30 general tissue types and 53 specific tissue types showed significant associations between brain tissues and headache, but not vascular tissues. This conclusion is different from the predominant theory of vascular etiology for migraine since our results suggest that brain function is closely related to broadly-defined headache. Combining all results from GWAS and FUMA together, it is clear that both neuronal and vascular factors are involved in the headache mechanism.

Consistent with previous studies from our group, we found strong evidence to support a shared genetic etiology of pain with psychological traits, indicating a vulnerability to depression and other negative mood states (McIntosh et al., 2016, van Hecke et al., 2017). In addition, we found a negative correlation with factors associated with a longer duration of education. Previous results have suggested that headache sufferers were more likely to have psychiatric disorders than healthy people (Mitsikostas and Thomas, 1999). In addition, a shared genetic link between migraine and depression has been identified (Stam et al., 2010). Further studies are needed to demonstrate the nature of these correlations and whether any directional ‘causal’ inferences can be drawn.

The SNP-based heritability for broadly-defined headache was 0.211 in our study, which is less than previously reported broad-sense heritability of migraine and tension-type headache of 40% (Russell et al., 2007, Schürks, 2012). This can be explained that the SNP-based heritability does not take gene-gene interactions and gene-environment interactions into account.

The Q-Q plot suggested that there are residual confounding factors between headache cases and controls which have not been adjusted for. Those could be factors associated with psychiatric and vascular disorders. We also noted there is a 90 degree upswing (around the *P* = 10^− 14^ level). This is due to the fact that we have a cluster of 1800 significant SNPs at this level (mainly located at the *LINC02210-CRHR1-MAPT* loci), which is over half of the total number of significant SNPs.

Using the CaTS power calculator, we had 80% power to identify SNP associations with a significance level of 5 × 10^− 8^, based on 74,461 cases and 149,312 controls, assuming an additive model, a minor disease allele frequency of 0.15, a genotypic relative risk of 1.05, and a prevalence of headache in the general population of 0.2 (a conservative assumption) (Skol et al., 2006).

The main limitation of our study is that the phenotyping was based on a specific pain-related question used by the UK Biobank cohort and was therefore broadly-defined. The specific pain question does not ask for any information about the headache severity, nor does it provide subgrouping opportunities such as differentiating migraine from tension-type headache or secondary headache types. In the future, when there is a new round of pain questionnaires to participants in the UK Biobank, more detailed and focused phenotyping would be generated.

We have identified 28 loci for broadly-defined headache as a single entity in a GWAS using the UK Biobank resource, including 14 loci that have previously been associated with migraine, and 14 loci that have not previously been associated with headache. This is the largest GWAS on headache in a single population so far. We also identified evidence that brain function is closely related to broadly-defined headache. In addition, we found several significant correlations with a number of psychological factors, suggesting that the genetic etiology of headache may also be related to these traits. These findings will not only contribute to the understanding of the causes of headache (and its subtypes) and its relationships with psychological disorders, it might also bring potential genetic target for drug treatment for patients with headache and psychological disorders.

The following are the supplementary data related to this article.

Supplementary Table 1 A summary of 3343 GWAS significant SNPs. Supplementary Table 1

Supplementary Table 2 Significant genes based on the gene analysis by MAGMA integrated in FUMA. Supplementary Table 2

Supplementary Table 3 Top 10 gene sets results by MAGMA integrated in FUMA. Supplementary Table 3

Supplementary Table 4 Tissue expression analysis on 30 general tissue types. Supplementary Table 4

Supplementary Table 5 Tissue expression analysis on 53 specific tissue types. Supplementary Table 5

Supplementary Table 6 Genetic correlations between headache and other phenotypes which reached significance of 0.05/234. Supplementary Table 6

## Funding Sources

This work was supported by the DOLORisk project [EU Horizon2020, grant number: 633491], the STRADL project [Wellcome Trust, grant number: 104036/Z/14/Z], and the Centre for Cognitive Ageing and Cognitive Epidemiology [Medical Research Council and Biotechnology and Biological Sciences Research Council, grant number: MR/K026992/1]. We are grateful for support from the Dr. Mortimer And Theresa Sackler Foundation. The funders had no role in study design, data collection, data analysis, interpretation, writing of the report.

## Conflicts of Interest

Ian Deary is a participant in UK Biobank. Other authors declare no conflict of interests.

## Authors' Contributions

WM conceptualized and designed the study, contributed to data analyzing, and wrote the manuscript. MA analyzed the data. HH contributed to the Table 2 and provided comments. ID provided essential comments. AM and BS organized the project and provided comments. All authors read and approved the final manuscript.

## Data Availability

All summary statistics can be shared upon request to non - commercial researchers.

The line indicates statistical significance of *P* = 0.001 (0.05/53).
