# Supplementary material for: A Genome-Wide Association Study Finds Genetic Associations with Broadly-Defined Headache in UK Biobank (N = 223,773)
Source: eBioMedicine. 2018 Jan 31;28:180–6. doi: 10.1016/j.ebiom.2018.01.023 (PMC5898025; doi:10.1016/j.ebiom.2018.01.023)
Supplement: Supplementary Table 3 — Top 10 gene sets results by MAGMA integrated in FUMA. [file mmc3.docx]

**Supplementary Table 3.** Top 10 gene sets results by MAGMA

| Number of Genes | Beta | Standard error | *P* | Full names of gene sets |
| --- | --- | --- | --- | --- |
| 1662 | 0.103 | 0.0256 | 2.81E-05 | positive_regulation_of_gene_expression |
| 6 | 1.58 | 0.396 | 3.25E-05 | mizukami_hypoxia_dn |
| 967 | 0.13 | 0.033 | 4.30E-05 | positive_regulation_of_transcription_from_rna_polymerase_ii_promoter |
| 1355 | 0.107 | 0.0283 | 8.22E-05 | neurogenesis |
| 185 | 0.277 | 0.0741 | 9.50E-05 | excitatory_synapse |
| 15 | 0.953 | 0.262 | 0.00013776 | apolipoprotein_binding |
| 20 | 0.952 | 0.262 | 0.00014088 | reflex |
| 51 | 0.493 | 0.138 | 0.00017085 | neuronal_postsynaptic_density |
| 167 | 0.255 | 0.0737 | 0.00027392 | jison_sickle_cell_disease_dn |
| 1134 | 0.109 | 0.0316 | 0.00028398 | nucleic_acid_binding_transcription_factor_activity |
