# Supplementary material for: A Genome-Wide Association Study Finds Genetic Associations with Broadly-Defined Headache in UK Biobank (N = 223,773)
Source: eBioMedicine. 2018 Jan 31;28:180–6. doi: 10.1016/j.ebiom.2018.01.023 (PMC5898025; doi:10.1016/j.ebiom.2018.01.023)
Supplement: Supplementary Table 4 — Tissue expression analysis on 30 general tissue types. [file mmc4.docx]

| Tissues | Beta | Standard error | *P* |
| --- | --- | --- | --- |
| Brain | 0.0416 | 0.00932 | 4.12E-06 |
| Blood.Vessel | 0.0406 | 0.0185 | 0.013996 |
| Pituitary | 0.023 | 0.0115 | 0.022878 |
| Uterus | 0.0381 | 0.0202 | 0.029401 |
| Fallopian.Tube | 0.034 | 0.0254 | 0.09009 |
| Colon | 0.0358 | 0.0284 | 0.10362 |
| Cervix.Uteri | 0.0349 | 0.0288 | 0.1131 |
| Testis | 0.0073 | 0.0072 | 0.15538 |
| Nerve | 0.00761 | 0.0168 | 0.32544 |
| Prostate | 0.00818 | 0.0236 | 0.36475 |
| Bladder | 0.000569 | 0.0252 | 0.491 |
| Ovary | 0.00016 | 0.0155 | 0.49588 |
| Esophagus | -0.00181 | 0.0291 | 0.5247 |
| Spleen | -0.00144 | 0.0109 | 0.55233 |
| Heart | -0.00256 | 0.0134 | 0.57607 |
| Lung | -0.00406 | 0.0172 | 0.59334 |
| Small.Intestine | -0.00363 | 0.0136 | 0.60546 |
| Adrenal.Gland | -0.00425 | 0.0149 | 0.61251 |
| Muscle | -0.00516 | 0.00931 | 0.71026 |
| Vagina | -0.0152 | 0.0219 | 0.75658 |
| Kidney | -0.0109 | 0.0151 | 0.76402 |
| Stomach | -0.0173 | 0.0226 | 0.77777 |
| Adipose.Tissue | -0.0202 | 0.0214 | 0.82736 |
| Thyroid | -0.0191 | 0.0182 | 0.85284 |
| Blood | -0.01 | 0.00857 | 0.87939 |
| Breast | -0.043 | 0.0287 | 0.93318 |
| Liver | -0.0133 | 0.00875 | 0.93543 |
| Skin | -0.0248 | 0.016 | 0.93961 |
| Pancreas | -0.0333 | 0.0129 | 0.99497 |
| Salivary.Gland | -0.0519 | 0.0175 | 0.99851 |
|  |  |  |  |

**Supplementary Table 4.** Tissue expression analysis on 30 general tissue types
