# Supplementary material for: A Genome-Wide Association Study Finds Genetic Associations with Broadly-Defined Headache in UK Biobank (N = 223,773)
Source: eBioMedicine. 2018 Jan 31;28:180–6. doi: 10.1016/j.ebiom.2018.01.023 (PMC5898025; doi:10.1016/j.ebiom.2018.01.023)
Supplement: Supplementary Table 5 — Tissue expression analysis on 53 specific tissue types. [file mmc5.docx]

| Tissues | Beta | Standard error | *P* |
| --- | --- | --- | --- |
| Brain_Cortex | 0.0409 | 0.00977 | 1.44E-05 |
| Brain_Frontal_Cortex_BA9 | 0.0388 | 0.00951 | 2.29E-05 |
| Brain_Anterior_cingulate_cortex_BA24 | 0.0368 | 0.0101 | 0.000132 |
| Brain_Cerebellar_Hemisphere | 0.0298 | 0.0083 | 0.000164 |
| Brain_Cerebellum | 0.031 | 0.00863 | 0.000166 |
| Brain_Caudate_basal_ganglia | 0.0378 | 0.011 | 0.000309 |
| Brain_Amygdala | 0.037 | 0.0111 | 0.000448 |
| Brain_Nucleus_accumbens_basal_ganglia | 0.0347 | 0.0105 | 0.000459 |
| Brain_Putamen_basal_ganglia | 0.0366 | 0.0111 | 0.000495 |
| Brain_Hippocampus | 0.037 | 0.0113 | 0.000509 |
| Brain_Hypothalamus | 0.0324 | 0.0114 | 0.002258 |
| Brain_Substantia_nigra | 0.0275 | 0.0125 | 0.013557 |
| Brain_Spinal_cord_cervical_c.1 | 0.0189 | 0.0123 | 0.061294 |
| Artery_Tibial | 0.0175 | 0.0152 | 0.124 |
| Pituitary | 0.011 | 0.0127 | 0.19337 |
| Colon_Sigmoid | 0.014 | 0.0196 | 0.23682 |
| Testis | 0.00348 | 0.00721 | 0.31476 |
| Uterus | 0.00604 | 0.0177 | 0.36613 |
| Artery_Aorta | 0.00495 | 0.0156 | 0.37562 |
| Esophagus_Gastroesophageal_Junction | 0.00362 | 0.0203 | 0.42909 |
| Cervix_Endocervix | 0.000829 | 0.0193 | 0.4829 |
| Esophagus_Muscularis | 0.000522 | 0.0193 | 0.48921 |
| Fallopian_Tube | -0.00413 | 0.02 | 0.58164 |
| Artery_Coronary | -0.00594 | 0.019 | 0.62285 |
| Cervix_Ectocervix | -0.0171 | 0.0217 | 0.78485 |
| Nerve_Tibial | -0.0154 | 0.0164 | 0.82529 |
| Spleen | -0.0102 | 0.0101 | 0.84215 |
| Heart_Atrial_Appendage | -0.014 | 0.0137 | 0.84781 |
| Prostate | -0.0202 | 0.0194 | 0.85064 |
| Cells_EBV.transformed_lymphocytes | -0.00715 | 0.00676 | 0.85473 |
| Colon_Transverse | -0.0174 | 0.0163 | 0.85791 |
| Small_Intestine_Terminal_Ileum | -0.0137 | 0.0119 | 0.87462 |
| Ovary | -0.018 | 0.0144 | 0.89442 |
| Lung | -0.0185 | 0.0143 | 0.9018 |
| Bladder | -0.0266 | 0.0196 | 0.91253 |
| Muscle_Skeletal | -0.0137 | 0.00935 | 0.92915 |
| Cells_Transformed_fibroblasts | -0.015 | 0.0101 | 0.93118 |
| Adrenal_Gland | -0.022 | 0.0142 | 0.93937 |
| Heart_Left_Ventricle | -0.0208 | 0.0123 | 0.95449 |
| Kidney_Cortex | -0.0244 | 0.0138 | 0.96204 |
| Whole_Blood | -0.0136 | 0.00754 | 0.96474 |
| Vagina | -0.0352 | 0.0182 | 0.97343 |
| Stomach | -0.0373 | 0.0186 | 0.97773 |
| Adipose_Visceral_Omentum | -0.0355 | 0.0171 | 0.98113 |
| Liver | -0.0181 | 0.00842 | 0.98423 |
| Thyroid | -0.0336 | 0.0155 | 0.98473 |
| Adipose_Subcutaneous | -0.0363 | 0.0165 | 0.98582 |
| Skin_Sun_Exposed_Lower_leg | -0.0269 | 0.011 | 0.99296 |
| Skin_Not_Sun_Exposed_Suprapubic | -0.028 | 0.011 | 0.99444 |
| Esophagus_Mucosa | -0.0278 | 0.0106 | 0.9956 |
| Breast_Mammary_Tissue | -0.0567 | 0.0209 | 0.99671 |
| Pancreas | -0.0409 | 0.0121 | 0.99965 |
| Minor_Salivary_Gland | -0.0562 | 0.0149 | 0.99992 |

**Supplementary Table 5.** Tissue expression analysis on 53 specific tissue types
