# Supplementary material for: A Genome-Wide Association Study Finds Genetic Associations with Broadly-Defined Headache in UK Biobank (N = 223,773)
Source: eBioMedicine. 2018 Jan 31;28:180–6. doi: 10.1016/j.ebiom.2018.01.023 (PMC5898025; doi:10.1016/j.ebiom.2018.01.023)
Supplement: Supplementary Table 6 — Genetic correlations between headache and other phenotypes which reached significance of 0.05/234. [file mmc6.docx]

| Trait 1 | Trait 2 | Category | Genetic correlation | Standard error | *P* |
| --- | --- | --- | --- | --- | --- |
| headache | Neuroticism | personality | 0.5018 | 0.0279 | 2.24E-72 |
| headache | Depressive symptoms | psychiatric | 0.5173 | 0.0361 | 1.60E-46 |
| headache | Years of schooling 2016 | education | -0.2834 | 0.0223 | 5.25E-37 |
| headache | Maternal age of first delivery | reproductive | -0.3191 | 0.0285 | 3.97E-29 |
| headache | Subjective well being | psychiatric | -0.3704 | 0.0419 | 9.51E-19 |
| headache | Insomnia | cognitive | 0.4208 | 0.0482 | 2.54E-18 |
| headache | College completion | education | -0.3199 | 0.0381 | 4.25E-17 |
| headache | Years of schooling 2013 | education | -0.2879 | 0.0344 | 6.41E-17 |
| headache | Years of schooling (proxy cognitive performance) | education | -0.2894 | 0.0349 | 1.12E-16 |
| headache | Neuroticism | personality | 0.521 | 0.0679 | 1.72E-14 |
| headache | Major depressive disorder | psychiatric | 0.3865 | 0.0573 | 1.57E-11 |
| headache | Number of children ever born | reproductive | 0.2268 | 0.034 | 2.56E-11 |
| headache | Intelligence | cognitive | -0.2142 | 0.0322 | 3.08E-11 |
| headache | PGC cross-disorder analysis | psychiatric | 0.1846 | 0.0395 | 3.05E-06 |
| headache | Schizophrenia | psychiatric | 0.1219 | 0.0275 | 9.08E-06 |
| headache | Sleep duration | sleeping | -0.1724 | 0.0423 | 4.51E-05 |
| headache | Platelet count | haemotological | 0.1419 | 0.035 | 5.02E-05 |
| headache | Triglycerides | lipids | 0.1514 | 0.0378 | 6.31E-05 |
| headache | Mothers age at death | aging | -0.2432 | 0.073 | 0.0009 |

**Supplementary Table 6.** Genetic correlations between headache and other phenotypes which reached significance of 0.05/234

Only phenotypes with *P* < 2.1 x 10^-4^ (0.05/234) were included in the Table.
